# 2021 Census to Census Coverage Survey Matching Results.

**DOI:** 10.23889/ijpds.v7i3.2018

**Published:** 2022-08-25

**Authors:** Charlie Tomlin, David Edwards

**Affiliations:** 1 ONS

The 2021 England and Wales Census was matched to the Census Coverage Survey (CCS). This was an essential requisite for estimating undercount in the Census. To ensure outputs could be produced within a year of census day, matching had to be completed in eight weeks.

We used the Data Access Platform to write efficient and accurate automatic matching algorithms. Probabilistic and associative matching methods were used to present candidate pairs that could not be automatically matched but likely consisted of many true matches. These were resolved by matchers in the clerical matching system. Active learning was used to iteratively improve probabilistic parameters as matches were made.

We also developed a pre-search algorithm that simplifies clerical matching by replacing clerical searching (here’s a record, can you find a match?) with clerical resolution (here are two records, do they match?).

As a result of our improvements, we increased our automatic matching rates from 70% (2011 Census) to 93% (2021 Census) for person matching and from 60% to 95% for household matching, without loss of accuracy. Precision and recall were estimated to both be 99.96% for person matching and 100% and 99.78% respectively for household matching. For both persons and households, the same trends seen in 2011 are seen here too; however, most of the biases have decreased and most match rates have increased.

Dual system estimation using the matched and unmatched records enabled us to estimate that coverage in the 2021 England and Wales Census was 97%.

The matching methodology implemented for the 2021 Census to CCS matching improved both person and household matching greatly when compared with the 2011 matching. The clerical matching system enabled clerical matching at scale and at pace. Thus, we successfully completed all clerical matching within our eight-week deadline.

